# Micro Photographs in Histology, Normal and Pathological

**Published:** 1876-09

**Authors:** 


Micro-Photographs in Histology, Normal and Pathological. By Carl Seiler, M.D., in conjunction with J. Gibbons Hunt and Joseph Richardson, M.D. Published monthly, by J. H. Coates & Co., 822 Chestnut street, Philadelphia. 50 cents a number, and $6 per annum.

We are in receipt of the June and July numbers of this publication, which contains each of them four plates, as follows: Longitudinal section of femur of human fœtus five months old; enchondroma from thigh; hyaline cartilage from thyroid cartilage of human larynx; tranverse section of dry bone; hepatic cells from liver of a fly; leukæmia of the liver; blood corpuscles of man and the ox; flat cells from mesentary of a cat.

